# Race, Prevalence of *POLE* and *POLD1* Alterations, and Survival Among Patients With Endometrial Cancer

**DOI:** 10.1001/jamanetworkopen.2023.51906

**Published:** 2024-01-17

**Authors:** Shuhua Zheng, Eric D. Donnelly, Jonathan B. Strauss

**Affiliations:** 1 Department of Radiation Oncology, Robert H. Lurie Comprehensive Cancer Center of Northwestern University, Chicago, Illinois

## Abstract

**Importance:**

Black patients with endometrial cancer (EC) in the United States have higher mortality than patients of other races with EC. The prevalence of *POLE* and *POLD1* pathogenic alterations in patients of different races with EC are not well studied.

**Objective:**

To explore the prevalence of and outcomes associated with *POLE* and *POLD1* alterations in differential racial groups.

**Design, Setting, and Participants:**

This retrospective cohort study incorporated the largest available data set of patients with EC, including American Association for Cancer Research Project GENIE (Genomics Evidence Neoplasia Information Exchange; 5087 participants), Memorial Sloan Kettering–Metastatic Events and Tropisms (1315 participants), and the Cancer Genome Atlas Uterine Corpus Endometrial Carcinoma (517 participants), collected from 2015 to 2023, 2013 to 2021, and 2006 to 2012, respectively. The prevalence of and outcomes associated with *POLE* or *POLD1* alterations in EC were evaluated across self-reported racial groups.

**Exposure:**

Patients of different racial groups with EC and with or without *POLE* or *POLD1* alterations.

**Main Outcomes and Measures:**

The main outcome was overall survival. Data on demographic characteristics, *POLE* and *POLD1* alteration status, histologic subtype, tumor mutation burden, fraction of genome altered, and microsatellite instability score were collected.

**Results:**

A total of 6919 EC cases were studied, of whom 444 (6.4%), 694 (10.0%), and 4869 (70.4%) patients were self-described as Asian, Black, and White, respectively. Within these large data sets, Black patients with EC exhibited a lower weighted average prevalence of pathogenic *POLE* alterations (0.5% [3 of 590 cases]) compared with Asian (6.1% [26 of 424]) or White (4.6% [204 of 4520]) patients. By contrast, the prevalence of *POLD1* pathogenic alterations was 5.0% (21 cases), 3.2% (19 cases), and 5.6% (255 cases) in Asian, Black, and White patients with EC, respectively. Patients with *POLD1* alterations had better outcomes regardless of race, histology, and *TP53* alteration status. For a total of 241 clinically annotated Black patients with EC, a composite biomarker panel of either *POLD1* or *POLE* alterations identified 7.1% (17 patients) with positive outcomes (1 event at 70 months follow up) in the small sample of available patients.

**Conclusions and Relevance:**

In this retrospective clinicopathological study of patients of different racial groups with EC, a composite biomarker panel of either *POLD1* or *POLE* alteration could potentially guide treatment de-escalation, which is especially relevant for Black patients.

## Key Points

**Question:**

What is the prevalence of *POLE* and *POLD1* pathogenic alterations and their association with outcomes in patients of different racial groups with endometrial cancer (EC)?

**Findings:**

In this retrospective cohort study of 6919 patients with EC with genetic and demographic information, *POLE* alterations were rare in Black patients, while *POLD1* appeared equally among racial groups. A composite biomarker panel of either *POLD1* or *POLE* alterations identified a higher number of patients with EC with good outcomes than using *POLE* alone.

**Meaning:**

These findings suggest that a composite biomarker panel of either *POLD1* or *POLE* alteration could potentially guide treatment de-escalation, especially relevant for Black patients.

## Teaser

This cohort study explores the prevalence of and outcomes associated with *POLE *and *POLD1 *alterations among patients of different races with endometrial cancer (EC).

## Introduction

There exists a large racial disparity in endometrial cancer (EC) mortality in the United States, with Black patients exhibiting a 5-year mortality rate approximately double that of White patients with EC.^1,2,3,4,5^ Poorer survival is associated with Black race, even after adjusting for surgery, histology, grade, age, stage at diagnosis, comorbid conditions, access to care, and socioeconomic factors.^5,6,7,8,9,10,11,12,13,14^ The Cancer Genome Atlas (TCGA) project identified 4 molecular subgroups of EC: DNA polymerase epsilon (*POLE*) ultra-altered, mismatch repair deficiency, copy-number (CN)–low, and CN-high.^15,16^ More recent studies utilized immunohistochemistry staining for p53, finding that this is a cost-effective surrogate for identification of the CN-high group, which has the poorest prognosis.^17,18,19,20,21^ *TP53* alteration is significantly more common in Black patients with EC than in White patients (75% vs 40%).^8,17,18,22^ The presence of other more favorable biomarkers may allow for tailored treatment de-escalation of treatment among patients with EC. This may be especially important for Black women, owing to their high risk of harboring a *TP53* alteration.

Pathogenic *POLE* alteration identified a cohort of patients with EC with favorable outcomes even with high-grade tumors, and it is actively studied in clinical trials, such as PORTEC-4a, RAINBO Blue, and TAPER, as a biomarker for treatment de-escalation.^23,24,25,26^ POLE is a critical protein with a 3′ to 5′ exonuclease domain involved in the recognition and excision of mismatched base pairs in the synthesis of the leading strand of DNA in the replication fork. Pathogenic alteration of *POLE* in the exonuclease domain in patients with EC is associated with ultra-alterations (>100 alterations per megabase), enhanced immune response, and favorable clinical outcome.^18,27,28,29,30,31,32,33,34^ Previous studies showed 5% to 10% of all ECs harbor pathogenic *POLE* alterations.^32,35,36,37^ Mirroring the role of *POLE*, DNA polymerase delta 1 (*POLD1*) is the major catalytic and proofreading subunit of DNA polymerase δ (Polδ), which is responsible for synthesis of the lagging DNA strand. *POLD1* alterations can also trigger accumulation of tumor mutation burden (TMB) and enhanced immune response.^38,39,40,41^ Previous studies showed *POLE* alterations are somatic while *POLD1* alterations are usually germline in EC.^42^ While *POLE* alterations have been well studied as a favorable biomarker and are currently under clinical trials for identification of treatment de-escalation, the prevalence of and outcomes associated with *POLD1* is unknown. This study aims to investigate the prevalence of *POLE* and *POLD1* by race and also evaluate the outcomes associated with *POLD1*.

## Methods

### Patient Selection

EC data sets in this study include the American Association for Cancer Research Project Genomics Evidence Neoplasia Information Exchange (AACR-GENIE; 5087 participants) version 13.1,^43^ Memorial Sloan Kettering–Metastatic Events and Tropisms (MSK-MET; 1315 participants), and TCGA Uterine Corpus Endometrial Carcinoma (TCGA-UCEC; 517 participants), which were collected from 2015 to 2023, 2013 to 2021, and 2006 to 2012, respectively. Briefly, AACR-GENIE collects clinical, next-generation cancer genomic sequencing (NGS) data obtained during routine medical practice in 19 top cancer centers globally. Alterations were detected via targeted gene sequencing panels based on cancer centers’ preference.^43^ Alterations in the MSK-MET data set were identified via hybridization capture–based deep NGS with a panel of all exons and selected introns of 341 key cancer genes.^44^ The TCGA-UCEC data set was sequenced with whole-exome sequencing based on Sanger Sequencing, and patients were selected if surgical resection was part of the treatment plan for patients who received no prior therapy.^15,45^ Based on the study by León-Castillo et al^25^ and others, the 5 most validated pathogenic *POLE* somatic alterations, including P286R, V411L, S297F, A456P, and S459F as well as less common pathogenic alterations including F367S, L424I, M295R, P436R, M444K, and D368Y, were selected to identify patients with EC and pathogenic alterations across 3 data sets.^18,25,32,35,36,37,42,46,47,48,49^ Cases with pathogenic *POLE* alterations were identified in the AACR-GENIE (189 cases), MSK-MET (66 cases), and TCGA-UCEC (50 cases) data sets. Clinicopathological data for these data sets were accessed via cBioPortal.^50,51^ Cases with *POLD1* alterations were also identified in the AACR-GENIE (249 cases), MSK-MET (78 cases), and TCGA-UCEC (40 cases) data sets. Self-reported racial distribution across these data sets was identified to study potential differential prevalence of pathogenic *POLE* and *POLD1* alterations. Categories for race followed those used in the data sets. Other racial groups, including Native American and Pacific Islander, were not included in the subgroup analysis due to limited sample sizes and inconsistent availability among data sets. Patients with no listed racial status were included in the unknown group; these patients were not included in the subgroup analyses in which racial information was required. This retrospective cohort study followed the Strengthening the Reporting of Observational Studies in Epidemiology (STROBE) reporting guideline and only used publicly available, deidentified data sets; therefore institutional review board approval or informed consent were not required per 45 CFR 46.

### OncoPrint Study

The OncoPrint study was performed using the cBioPortal platform.^50,51^ Briefly, EC cases from MSK-MET were subgrouped by TMB into those with less or equal than 100 (TMB ≤100; 1264 cases) and those larger than 100 (TMB >100; 48 cases). Cases with TMB greater than 100 were aligned from highest TMB to lowest TMB with the height of corresponding columns representing the value of TMB. Corresponding genetic alteration status of *POLE* and *POLD1* were aligned and color-coded.

### Alteration Diagram

An alteration diagram was generated in cBioPortal. *POLE* alterations, including P286R, V411L, S297F, A456P, S459F, F367S, L424I, M295R, P436R, M444K, and D368Y, were selected to identify patients with EC and pathogenic alterations. Diagram circles were color-coded to represent pathogenic alterations. Three domains of the *POLE* gene, including polymerase exonuclease (DNA-pol_B_exo1), DNA polymerase (DNA_pol_B), and domain of the unknown function (DUF1744) were labeled.

### Statistical Analysis

Statistical analyses were performed using the Prism version 8.0 (GraphPad). Overall survival (OS) was expressed as the number of months from diagnosis to death, and survival probabilities were calculated using the Kaplan-Meier method. Differences between survival rates were tested with the log-rank test. Comparison of the probability of unpaired events in each subgroup was conducted with Mann-Whitney test. A 2-tailed *P* < .05 was considered statistically significant.

## Results

### Prevalence of Pathogenic *POLE* Alterations by Race

**Figure 1.**
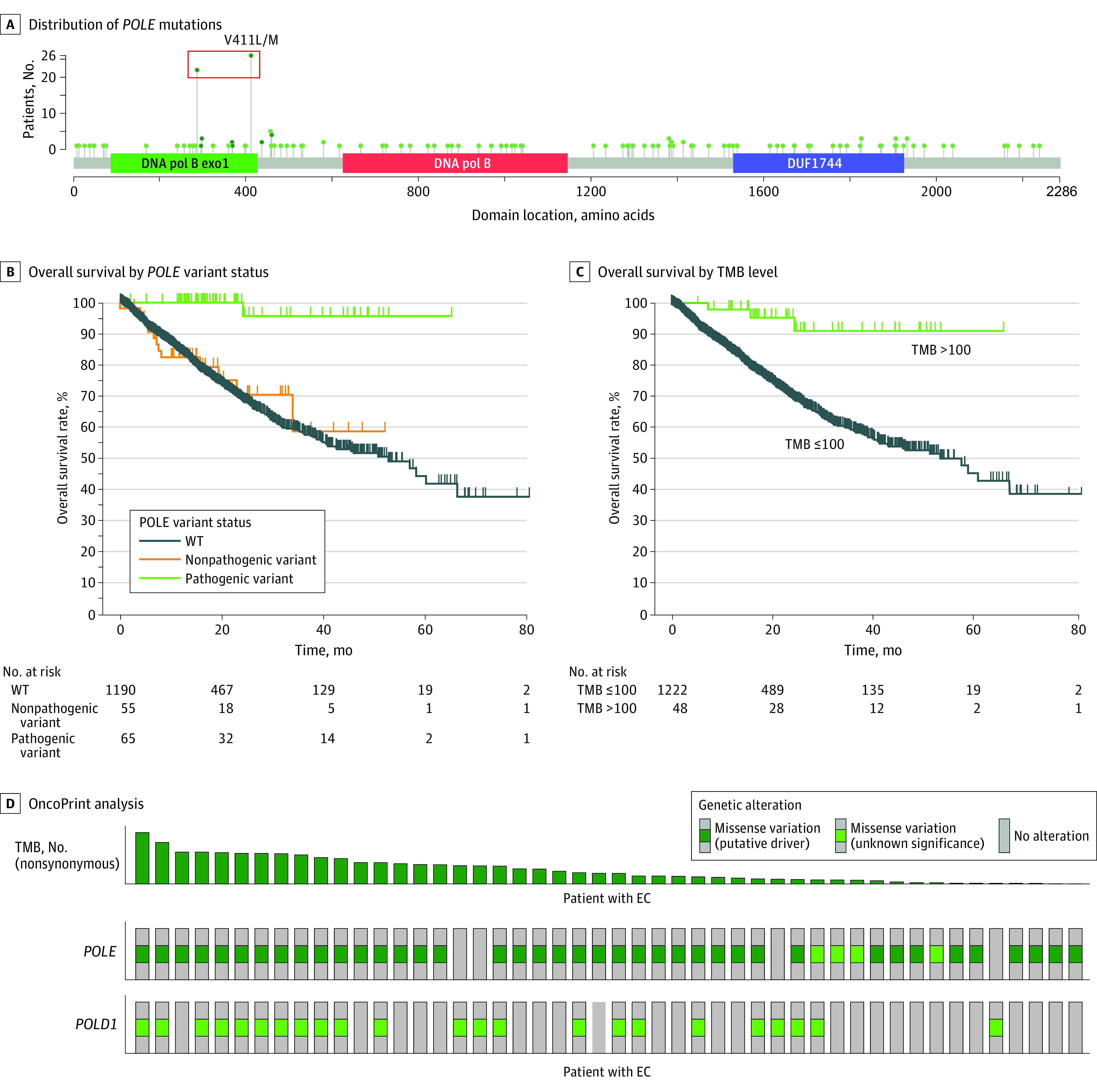
Distribution of *POLE* and *POLD1* Alterations and Outcomes Evaluation of Tumor Mutation Burden (TMB) A, Distribution of *POLE* alterations in patients with endometrial cancer (EC). Most common alterations included P286R (71 cases) and V411L/M (60 cases), accounting for 69.3% of all the cases with pathogenic alterations (red box). B, Kaplan-Meier survival analysis of the Memorial Sloan Kettering–Metastatic Events and Tropisms data set between patients with EC and pathogenic *POLE* alterations, nonpathogenic *POLE* alterations, and *POLE*-wildtype (WT) (*P* < .001). C, Kaplan-Meier analysis of patients with EC with TMB greater than 100 and those with TMB of 100 or less (*P* < .001). D, OncoPrint analysis of *POLE* and *POLD1* alterations in patients with EC with TMB greater than 100. DNA pol B indicates DNA polymerase; DNA pol B exo1, DNA polymerase exonuclease; and DUF1744, domain of the unknown function.

A total of 6919 EC cases were included in this retrospective cohort study, of whom 444 (6.4%), 694 (10.0%), and 4869 (70.4%) patients were self-described as Asian, Black, and White, respectively. First, we studied the prevalence of *POLE* alterations within different racial groups in the largest available data sets that had NGS, including AACR-GENIE (5087 participants) and MSK-MET (1315 participants).^43^ In the AACR-GENIE data set, a total of 330 (6.7%), 456 (9.2%), 3549 (71.8%), and 242 (4.9%) patients were self-described as Asian, Black, White, or unknown, respectively. Overall, 189 (3.7%) had pathogenic *POLE* alterations, with P286R (71 cases) and V411L/M (60 cases) accounting for 69.3% of all the cases with pathogenic alterations (Figure 1A, red box). Other pathogenic alterations included S297F (10 cases), A456P (18 cases), S459F (10 cases), F367S (6 cases), L424I (1 cases), M295R (1 case), P436R (8 cases), M444K (2 cases), and D368Y (2 cases). Overall, 19 (5.8%), 1 (0.2%), and 152 (4.3%) Asian, Black, and White patients with EC had pathogenic *POLE* alterations (Table). We then studied the MSK-MET data set, in which 94 (7.1%), 134 (10.2%), 971 (73.8%), and 37 (2.8%) patients were self-described as Asian, Black, White, and unknown, respectively. Among them, 7 (7.4%), 2 (1.5%), and 52 (5.4%) Asian, Black, and White patients with EC had pathogenic *POLE* alterations (Table). In sum, only 3 of 590 cases (0.5%) among Black patients with EC from AACR-GENIE and MSK-MET data sets had a pathogenic *POLE* alteration, compared with an average of 6.1% for Asian patients with EC or 4.5% for White patients, respectively.

**Table.** Prevalence of Pathogenic *POLE* and *POLD1* Alterations in Black, White, and Asian Patients With Endometrial Cancer in the AACR-GENIE and MSK-MET Data Sets

Data set	Patients, No. (%)^a^		
	Asian	Black	White
AACR-GENIE			
* POLE*	19 (5.8)	1 (0.2)	152 (4.3)
* POLD1*	15 (4.5)	13 (2.8)	196 (5.5)
MSK-MET			
* POLE*	7 (7.4)	2 (1.5)	52 (5.4)
* POLD1*	6 (6.4)	6 (4.5)	59 (6.1)

Abbreviations: AACR-GENIE, American Association for Cancer Research Project Genomics Evidence Neoplasia Information Exchange; MSK-MET, Memorial Sloan Kettering–Metastatic Events and Tropisms.

^a^ There were 330 Asian patients, 456 Black patients, and 3549 White patients in the AACR-GENIE data set and 94 Asian patients, 134 Black patients, and 971 White patients in the MSK-MET data set.

### Clinical and Genomic Features of Pathogenic *POLE* Alteration in EC

Survival data were not available in the AACR-GENIE data set. Therefore, OS analysis was performed in the MSK-MET data set. We found patients with EC and pathogenic *POLE* alterations (66 patients) had superior outcomes (1 event at 65 months follow-up; median OS not reached), whereas patients with nonpathogenic *POLE* alterations (55 patients; median OS not reached) and *POLE*-wildtype alterations (1190 patients; median OS, 52.86 months; 95% CI, 40.81 months to not applicable) had similar OS rates (*P* < .001) (Figure 1B). Histopathological study in the MSK-MET data set further showed 100% of patients with pathogenic *POLE *alterations were classified as uterine endometroid carcinoma (UEC) vs 87.27% and 60.36% for patients with nonpathogenic alterations and wildtype alterations, respectively (eTable in Supplement 1). Tumors with pathogenic *POLE *alterations showed a median microsatellite instability (MSI) score of 17.14, corresponding to 61% MSI instability, compared with scores of 0.31 (11.7% MSI instability) and 0.17 (6.1% MSI instability) for wild-type tumors and tumors with nonpathogenic *POLE *alterations, respectively (eTable in Supplement 1). Ultra-alteration (TMB >100) was mainly identified in the groups with pathogenic *POLE *alterations (eTable in Supplement 1). These data suggested, as other studies have, that only pathogenic *POLE* alterations were associated with the classic *POLE* markers. We also found 48% and 92% of cases with TMB greater than 100 exhibited *POLD1* and *POLE* missense variants, respectively (Figure 1D). OS analysis showed that the 48 patients with EC and TMB greater than 100 had better survival rates than those with TMB of 100 or less (1222 patients) (*P* < .001) (Figure 1C). We then further studied prevalence of and outcomes associated with *POLD1* in patients with EC.

### Prevalence of *POLD1* Alterations by Race

We studied the prevalence of *POLD1* alterations on the AACR-GENIE and MSK-MET data sets, the largest data sets with NGS. In the AACR-GENIE data set, 15 (4.5%), 13 (2.8%), and 196 (5.5%) Asian, Black, and White patients with EC exhibited *POLD1* alterations (Table). In the MSK-MET data set, 6 (6.4%), 6 (4.5%), and 59 (6.1%) of Asian, Black, and White patients with EC had *POLD1* alterations (Table). By weighted average, the prevalence of patients with EC and *POLD1* alterations was 5.0% (21 cases) in Asian, 3.2% (19 cases) in Black, and 5.6% (255 cases) in White patients.

### Presence of Coexisting *POLE* and *POLD1* Pathogenic Alterations

**Figure 2.**
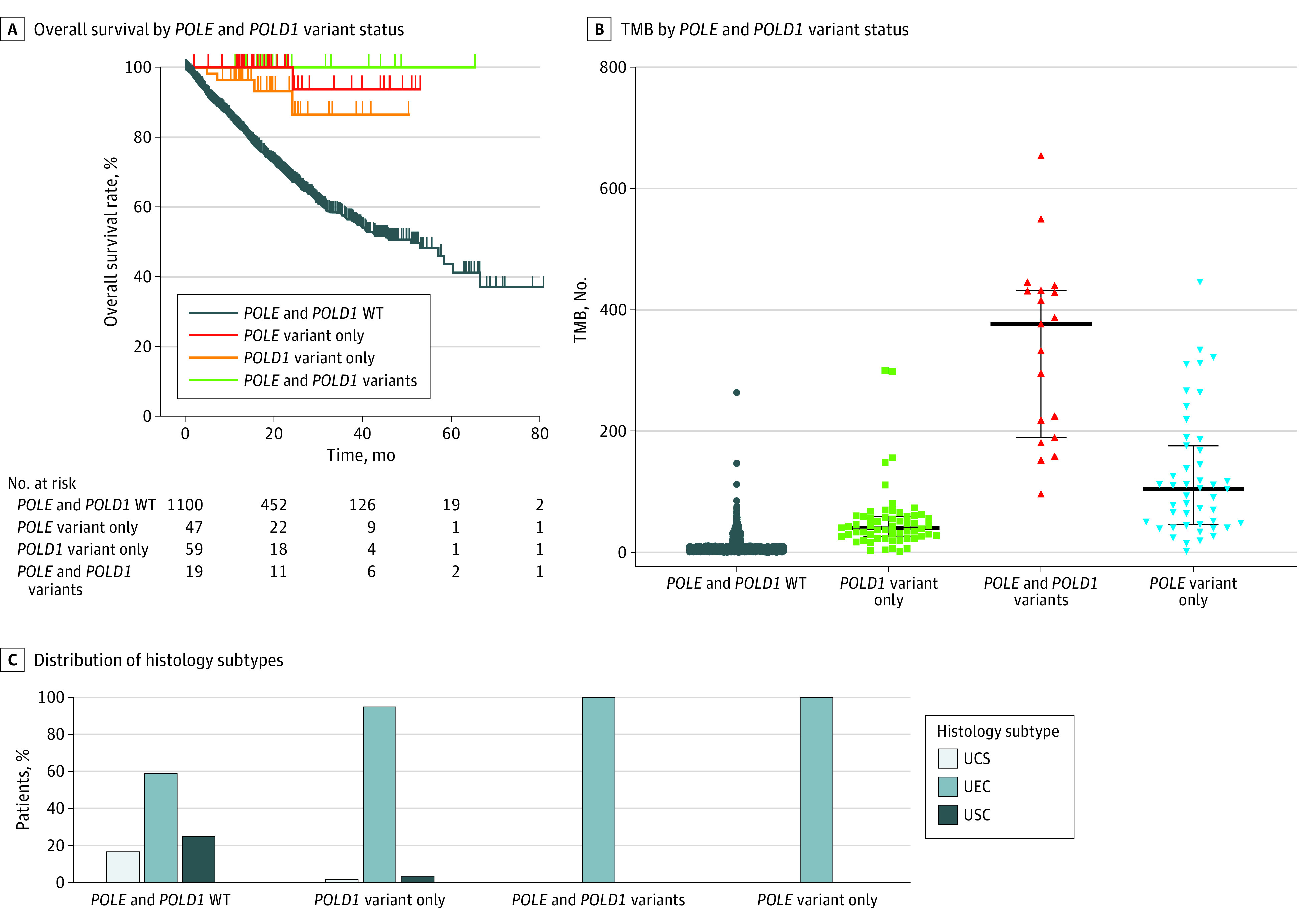
Identification of *POLE* and *POLD1* Pathogenic Alterations in Endometrial Cancer A, Overall survival analysis of patients with endometrial cancer with *POLE* and *POLD1* wildtype (WT), *POLE *variant only, both *POLE* and *POLD1* variant, and *POLD1* variant alone(*P* = .20). B, Tumor mutation burden (TMB) of cohorts with *POLE* and *POLD1* WT, POLE variant only, *POLE* and *POLD1* variants, and POLD1 variants only (*P* < .001). The horizontal lines indicate the median; whiskers, IQR; and the dots, each sample’s TMB. C, Distribution of histology subtypes between cohorts of *POLE* and *POLD1* WT, *POLE* variant only, *POLE* and *POLD1*, and *POLD1* variant only (*P* < .001). UCS indicates uterine carcinosarcoma; UEC, uterine endometroid carcinoma; USC, uterine serious carcinoma.

We found that in the AACR-GENIE data set, 27% of *POLE-*altered cases (51 of 189) coexisted with *POLD1* alterations (Figure 2A). OS analysis in the MSK-MET data set showed similarly favorable survival for *POLE* and *POLD1*–altered vs *POLE-*altered vs *POLD1*-altered tumors (*P* = .20) (Figure 2A). Tumors with both *POLE* and *POLD1* alterations showed higher TMB than either alteration alone, with a median (IQR) TMB of 374.4 (189.4-431.6), compared with 44.1 (26.0-58.8) and 105.5 (45.8-167.4) for *POLD1* only and *POLE* only, respectively (*P* < .001) (Figure 2B). Histologically, 100% of cases with both *POLE* and *POLD1* alterations and *POLE* alterations alone were UEC vs 60% for those with wildtype (673 of 1140) (*P* < .001) (Figure 2C).

### Clinical and Genomic Features of Pathogenic *POLD1* Alteration in EC

**Figure 3.**
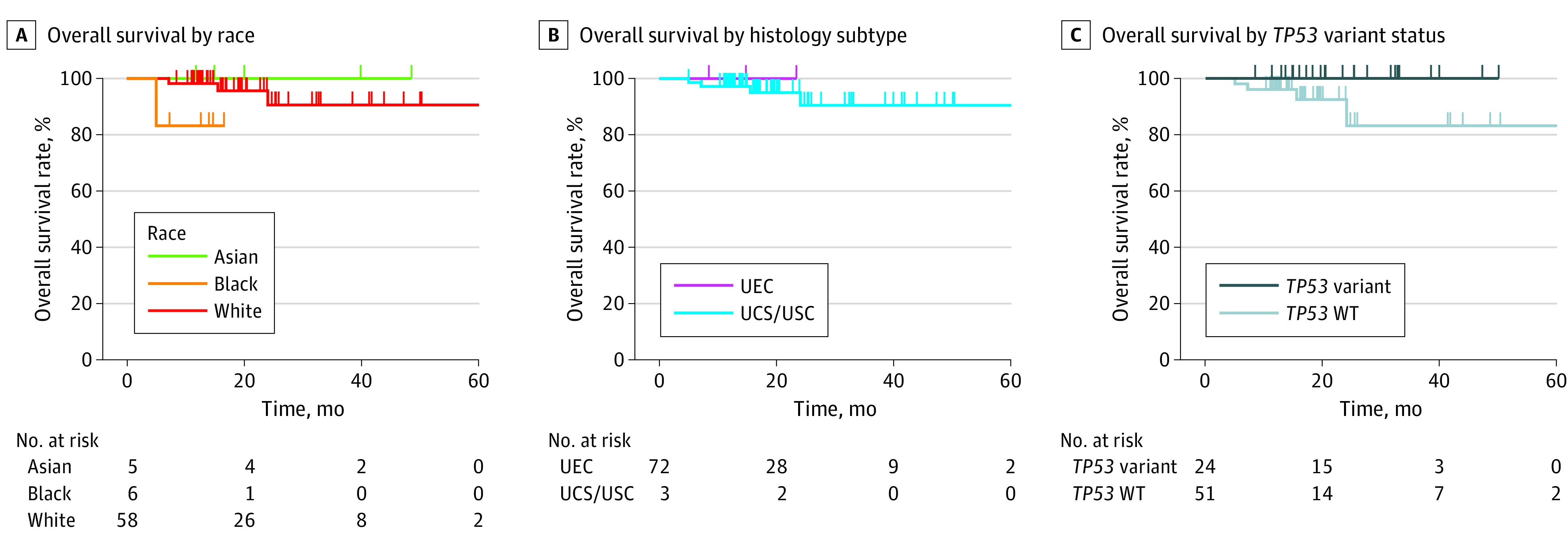
*POLD1* Alterations and Outcomes Among Patients With Endometrial Cancer A, Kaplan-Meier analysis of *POLD1-*altered endometrial cancer among patients of different race groups (*P* = .13). B, Kaplan-Meier analysis by histology subtypes (*P* = .74). C, Kaplan-Meier analysis by *TP53* alteration status (*P* = .10). UCS indicates uterine carcinosarcoma; UEC, uterine endometroid carcinoma; USC, uterine serious carcinoma; WT, wild-type.

In the MSK-MET data set with *POLD1* alterations, patients with EC had excellent outcomes regardless of race (5 Asian patients; 6 Black patients,; 58 White patients; *P* = .13), histology (72 patients with UEC and 3 with uterine serious carcinoma or uterine carcinosarcoma; *P* = .74), or *TP53* altered status (24 with *TP53* altered; 51 with *TP53* wildtype; *P* = .10) (Figure 3). Compared with TCGA/Proactive Molecular Risk Classifier for Endometrial Cancer EC risk classification algorithm, the composite biomarker panel of *POLE* or *POLD1* alterations identified 28.6% more Asian, 350% more Black, and 86.5% more White patients with EC with good outcomes than using *POLE* alone (9 of 94 [9.6%] vs 7 of 94 [7.4%] Asian patients; 7 of 134 [6.0%] vs 2 of 134 [1.5%] Black patients; 97 of 971 [10%] vs 52 of 971 [5.4%] White patients) (eFigure in Supplement 1).^16^ Due to the paucity of Black patients with EC and low prevalence of pathogenic *POLE* alterations, such subgroup analysis was not feasible in the MSK-MET data set as a single cohort.

### Integrating *POLD1* Alterations for Prognostication of Black Patients With EC

**Figure 4.**
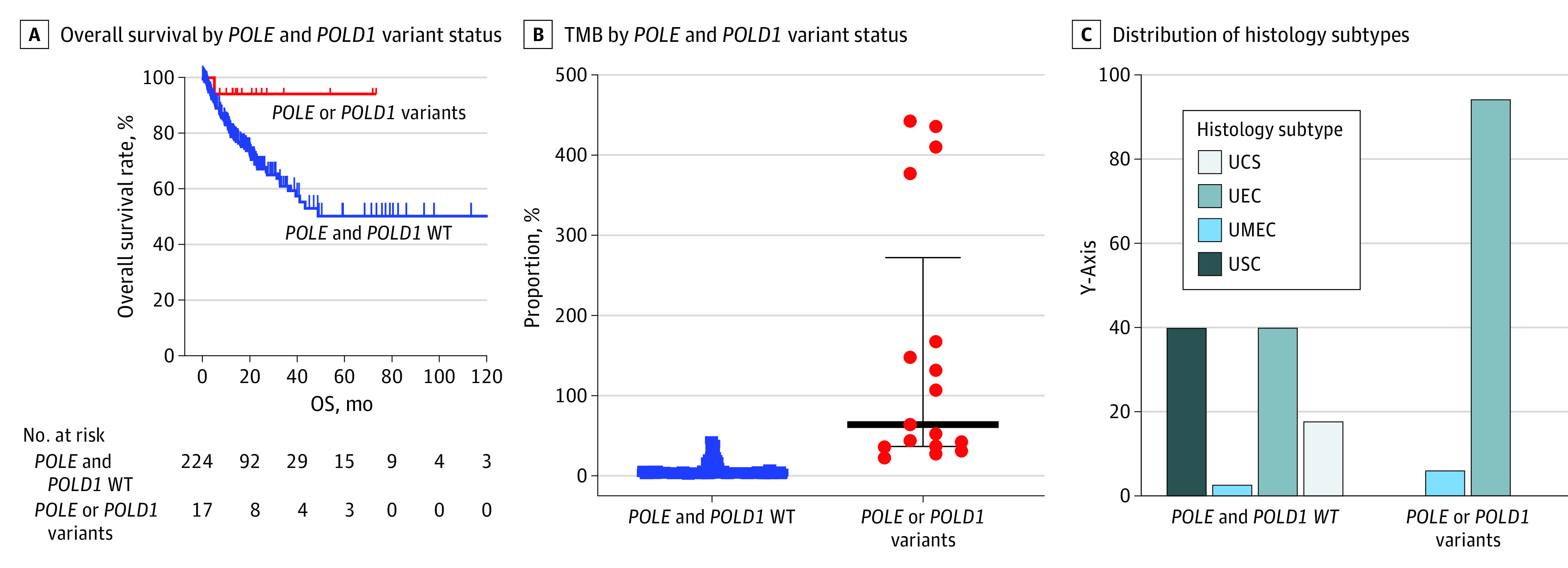
*POLE* and *POLD1* in Black Patients With Endometrial Cancer A, Kaplan-Meier analysis of Black patients with endometrial cancer and either *POLE* or *POLD1* alteration vs those with wildtype (WT) (*P* = .046). B, Scatterplot analysis of tumor mutation burden (TMB) for Black patients with EC and *POLE* or *POLD1* alteration vs *POLE* and *POLD1* WT (median TMB: 64.13 vs 3.46, *P* < .001). The horizontal lines indicate the median; whiskers, IQR; and the dots, each sample’s TMB. C, Distribution of histology subtypes between patients with *POLE* or *POLD1* altered vs *POLE* and *POLD1* WT. UCS indicates uterine carcinosarcoma; UEC, uterine endometroid carcinoma; UMEC, uterine mixed endometrial carcinoma; USC, uterine serious carcinoma.

Due to limited numbers of Black patients with EC and pathogenic *POLE* or *POLD1* alterations with survival data available, we combined TCGA-UCEC and MSK-MET data sets and identified 241 Black patients with EC with required clinicopathological information. Of these, 17 Black patients with EC and either *POLE* alterations (7 patients) or *POLD1* alterations (10 patients) were identified (Figure 4A). OS analysis showed only 1 event with as long as 70 months of follow-up in the cohort with either *POLE1* or *POLD1* alterations vs median OS of 48.8 months (95% CI, 48.8 months to not applicable) for the wildtype cohort of 224 patients (*P* = .046) (Figure 4A). Cases with either *POLE* or *POLD1* alterations had significantly higher TMB than those with wildtype (median [IQR], 64.13 [36.1-167.36] vs 3.46 [1.77-6.05]; *P* < .001) (Figure 4B). Histologically, 94% of cases (16 cases) with either *POLE* or *POLD1* alterations were UEC vs 40% (89 cases) for those with wildtype (*P* < .001) (Figure 4C).

## Discussion

In this large genomic and demographic cohort study of patients with EC, pathogenic alterations in both *POLE* and *POLD1* were associated with favorable outcomes in the cohort as a whole and in Black patients specifically. Whereas *POLE* alterations were quite uncommon in Black patients with EC, *POLD1* alterations were more evenly distributed across racial groups. Specifically, only 0.5% of Black patients with EC from AACE-GENIE and MSK-MET data sets had any known pathogenic *POLE* alterations. By contrast, 3.6% of Black patients with EC had pathogenic *POLD1* alterations. Therefore, optimal molecular risk assessment should incorporate both findings, and this dual biomarker strategy could be especially important for Black patients for whom *POLE* alterations are rare.

*POLE* and *POLD1* are essential for proofreading in DNA replication, the alteration of which will cause DNA hyperalteration and often indicate better response to immunotherapy across many cancer types.^40,52,53,54^ The expression levels of *POLD1* have been incorporated in prognosis of many cancers that were not treated with immunotherapy.^55,56^ In the absence of immunotherapy, *POLD1* is not always associated with favorable prognosis.^40^ However, in the present study, the presence of *POLD1* alteration in EC appeared to be associated with excellent clinical outcomes regardless of racial, histological, and *TP53* alteration status disparities.

Previous studies identified that 75% of Black patients with EC had *TP53* alteration compared with 40% for White patients with EC.^8,17,18,22^ *TP53* is used as a biomarker to identify high-risk patients for treatment intensification, and *POLE* sequencing is rapidly entering clinical practice for treatment de-escalation.^23,24^ Given the high prevalence of *TP53* alteration and low prevalence of pathologic *POLE* alterations, Black patients with EC have a very high likelihood of being classified in a poor prognostic group with an indication for therapeutic escalation based on current molecular classification system.^17,18^ The use of a composite biomarker of either *POLE* or *POLD1* alterations as an alternative may identify more patients, especially Black patients, who may benefit from treatment de-escalation.

### Limitations

This study has limitations, including its retrospective nature and scarcity of available clinical and genetic data sets for Black patients with EC. Therefore, this study highlights the need for enrollment of Black patients with EC in molecular studies. Another limitation is that this study did not include socioeconomics, disease stage, and treatment variables of potential interest for prognosis. Similarly, OS is an important outcome variable, but other outcomes, such as disease progression survival and endometrial cancer–specific survival, were not investigated in this study owing to limitations in data sets. *POLD1* is not well studied in patients with EC overall. This study included all potential pathologic *POLD1* variations except inframe variants, which may potentially be overinclusive. Furthermore, different sequencing techniques used across different data sets and a low number of Black patients with EC may generate variations of *POLE* and *POLD1* alteration prevalence between study cohorts.

## Conclusions

In this study, *POLD1* alterations appeared to be associated with excellent prognosis among patients with EC. Pathogenic *POLE* alterations were rare in Black patients with EC. In contrast, Black patients with EC were approximately equally likely as patients of other racial groups to have *POLD1* alterations. A composite biomarker panel of either *POLD1* or *POLE* alteration could guide identification of appropriate candidates for treatment de-escalation, and this finding is especially relevant for Black patients with EC.

## Notes

Supplement 1. **eTable.** Clinical, Genetic, and Pathological Features of Patients With EC From AACR-GENIE Data Set **eFigure. **OS Analysis Evaluating the Potential of Incorporating POLD1 in the Current TCGA/ProMisE Endometrial Cancer Risk Classification Algorithm

Supplement 2.
Data Sharing Statement
