# Supplementary material for: Race, Prevalence of POLE and POLD1 Alterations, and Survival Among Patients With Endometrial Cancer
Source: JAMA Netw Open. 2024 Jan 17;7(1):e2351906. doi: 10.1001/jamanetworkopen.2023.51906 (PMC10794941; doi:10.1001/jamanetworkopen.2023.51906)
Supplement: Supplement 1. — eTable. Clinical, Genetic, and Pathological Features of Patients With EC From AACR-GENIE Data Set eFigure. OS Analysis Evaluating the Potential of Incorporating POLD1 in the Current TCGA/ProMisE Endometrial Cancer Risk Classification Algorithm [file jamanetwopen-e2351906-s001.pdf]

Supplemental Online Content

Zheng S, Donnelly ED, Strauss JB. Race, prevalence of *POLE* and *POLD1* alterations, and survival among patients with endometrial cancer. *JAMA Netw Open.* 2024;7(1):e2351906. doi:10.1001/jamanetworkopen.2023.51906

eTable. Clinical, Genetic, and Pathological Features of Patients With EC From AACR-GENIE Data Set

eFigure. OS Analysis Evaluating the Potential of Incorporating POLD1 in the Current TCGA/ProMisE Endometrial Cancer Risk Classification Algorithm

This supplemental material has been provided by the authors to give readers additional information about their work.

© 2024 Zheng S et al. *JAMA Network Open.*

eTable. Clinical, Genetic, and Pathological Features of Patients With EC From AACR-GENIE Data Set

	**POLE_WT (n=1193)**	**POLE_MT_Non-Path. (n=55)**	**POLE_MT_Path. (n=66)**	***p*-Value**
Age Median (IQR)	65.51 (58.96 to 71.17)	65.85 (57.22 to 69.4)	56.03 (50.11 to 61.83)	<0.001
Met. Count Median (IQR)	2 (0 to 5)	2 (0 to 4)	0 (0 to 1)	<0.001
FGA Median (IQR)	0.06 (0 to 0.27)	0.03 (0 to 0.09)	0 (0 to 0)	<0.001
MSI score Median (IQR)	0.31 (0 to 1.84)	17.14 (3.47 to 27.22)	0.17 (0 to 0.72)	<0.001
TMB Median (IQR)	5.19 (3.46 to 8.65)	31.99 (19.89 to 45.83)	132.3 (65.72 to 295.73)	<0.001
UEC No. (%)	720 (60.36)	48 (87.27)	66 (100)	<0.001
UCS No. (%)	189 (15.8)	3 (5.45)	0 (0)	<0.001
USC No. (%)	284 (23.8)	4 (7.27)	0 (0)	<0.001
Ultramutation No. (%)	4 (0.3)	4 (7.3)	45 (68.2)	<0.001

Wildtype POLE (POLE_WT), non-pathogenically mutated POLE (POLE_MT_Non-Patho.), POLE pathogenically mutated (POLE_MT_Path.). TMB, tumor mutation burden; Age, years; MSI, microsatellite instability; UEC, uterine endometroid carcinoma; USC, uterine serious carcinoma; UCS, uterine carcinosarcoma; FGA, fraction of genome altered; IQR, interquartile range.

© 2024 Zheng S et al. *JAMA Network Open.*

eFigure. OS Analysis Evaluating the Potential of Incorporating *POLD1* in the Current TCGA/ProMisE Endometrial Cancer Risk Classification Algorithm

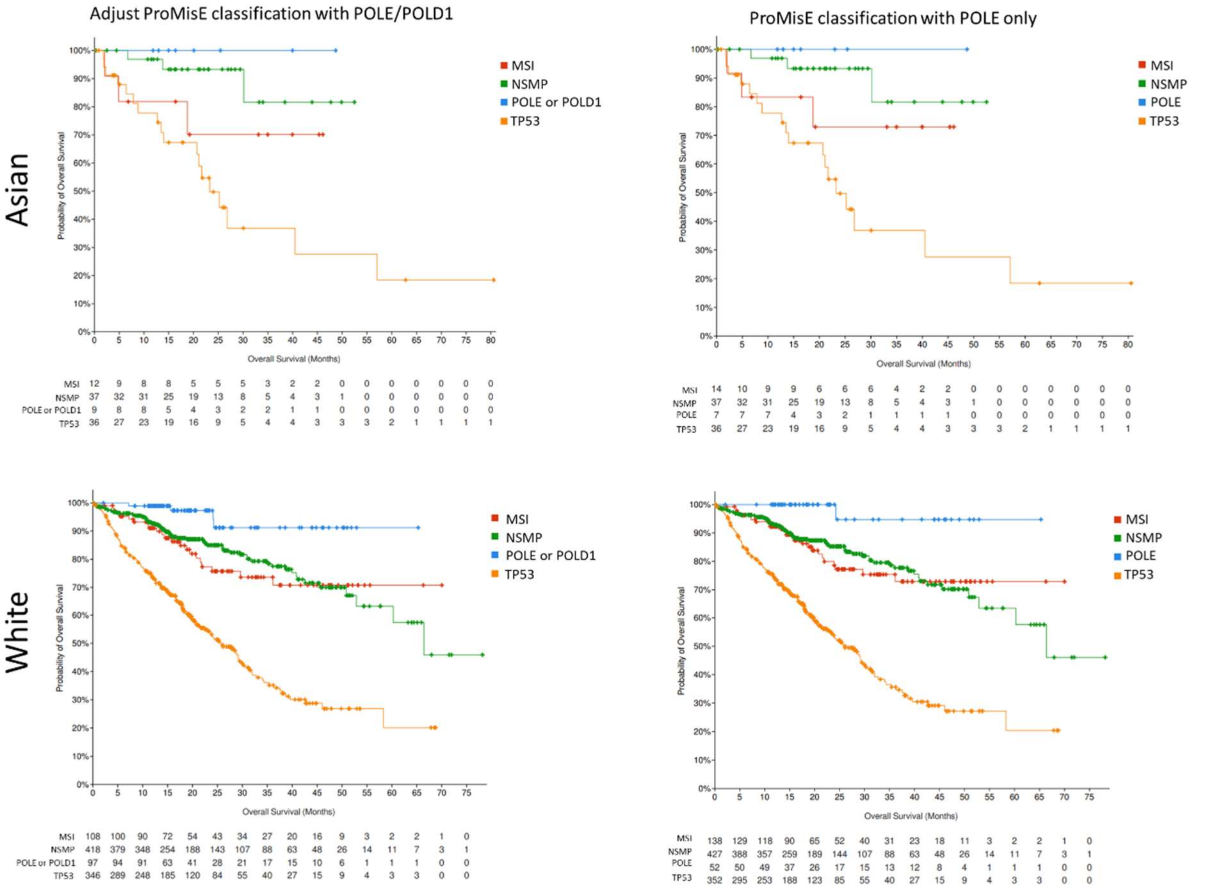

OS analysis of the combined POLE/POLD1 biomarker panel in identifying Asian and White EC patients in the MSK-MET dataset. Patients were classified based on the classic TCGA/ProMisE risk classification in the Right panels. Left panels incorporated POLE or POLD1 as a biomarker panel in identifying low risk patients. Patients numbers are provided as a chart at the bottom of each survival analysis. MSI: microsatellite instability; NSMP: No Specific Molecular Profile.

© 2024 Zheng S et al. *JAMA Network Open.*
