# Supplementary material for: Race, Prevalence of POLE and POLD1 Alterations, and Survival Among Patients With Endometrial Cancer
Source: JAMA Netw Open. 2024 Jan 17;7(1):e2351906. doi: 10.1001/jamanetworkopen.2023.51906 (PMC10794941; doi:10.1001/jamanetworkopen.2023.51906)
Supplement: Supplement 2. — Data Sharing Statement [file jamanetwopen-e2351906-s002.pdf]

Data Sharing Statement

Zheng. Race, Prevalence of POLE and POLD1 Alterations, and Survival Among Patients With Endometrial Cancer. *JAMA Netw Open*. Published January 17, 2024.
doi:10.1001/jamanetworkopen.2023.51906

Data
Data available: Yes
Data types: Data dictionary
How to access data: https://www.cbioportal.org/study/summary?id=msk_met_2021
https://genie.cbioportal.org/login.jsp
When available: With publication

Supporting Documents
Document types: None

Additional Information
Who can access the data: Follow cBioPortal data sharing policy
Types of analyses: Follow cBioPortal data sharing policy
Mechanisms of data availability: Follow cBioPortal data sharing policy
